# Continuous monitoring of radiation emissions from ^131^I thyroid cancer ablation subjects: development of a novel radiation detector system and measurement of effective retention half-time in 250 subjects

**DOI:** 10.22038/aojnmb.2025.88612.1638

**Published:** 2026

**Authors:** Dale L Bailey, Afsaneh Lahooti, Kathy P Willowson, Brian H C Shin, Carl Muñoz-Ferrada

**Affiliations:** 1 Department of Nuclear Medicine, Royal North Shore Hospital, Sydney, Australia; 2 Faculty of Medicine & Health, University of Sydney, Sydney, Australia; 3 Nanoscale Organization and Dynamics Group, School of Science, Western Sydney University, Sydney, Australia; 4 Institute of Medical Physics, Faculty of Science, University of Sydney, Sydney, Australia; 5 Globalsonics Research Pty Ltd, Sydney, Australia

**Keywords:** Radiation exposure, Iodine-131, Thyroid ablation, Retention, Detector A B S T R A C T

## Abstract

**Objectives(s):**

To report methodology that has been developed to provide real-time monitoring of radiation emissions from subjects treated with radionuclide therapies and summarise the radioiodine retention profiles of 250 subjects treated for differentiated thyroid cancer with ^131^I.

**Methods:**

A small ceiling-mounted radiation detector for continuously monitoring the exposure rate in the radiation isolation rooms has been developed. Measurements were made every minute after administration of 1-6 GBq of ^131^I over the one to three days typical inpatient admission. The data are saved in text format and have been fitted with a mono-exponential curve to measure retention half time.

**Results:**

The average effective retention half time (t_½_ (Eff)) for all subjects was 11.9±3.2 hrs (range: 5.0–23.1 hrs; n=250). Over 90% of the subjects had their serum TSH levels increased by injection of recombinant human TSH prior to treatment. Average retention half-time was found to be less in subjects lower than 55 year of age (t_½_ (Eff)=11.5 hrs) compared to those 55 or older (t_½_ (Eff)=14.4 hrs) (P=0.0007).

**Conclusions:**

Despite the subjects, being free to move around the isolation room during admission and thus changing the source-detector geometry markedly, the system has been able to characterise their retention profiles after radioiodine treatment. These real-time measurements have applications in planning therapy and monitoring the subjects during their admission to the hospital and can be used for “live” updates for all staff as well as providing insights into the fate of radioiodine in the body.

## Introduction

 The well-established standard management of the individual with differentiated thyroid cancer in most institutions is to have definitive surgery followed by an oral dose of radioiodine (^131^I; t_½_=8 days) with the aim of ablating any remnant thyroid or cancer cells. Considerable debate exists as to the appropriate risk/benefit of the amount of radioiodine to administer, as well as the intent, with choices generally ranging from 1 GBq to 6 GBq (1). In many jurisdictions, these amounts of administered radioiodine require isolation of the subject from the public for a period of time and so they are admitted as inpatients to the hospital facility. To maximise the amount of radioiodine taken up by the thyroid cells, to have the best clinical outcome, the subjects generally have an intervention prior to treatment to raise their serum TSH levels to drive more of the radioiodine into the cells thus delivering the highest possible radiation dose. This can either take the form of withdrawal of the thyroid hormone thyroxine (T4) or, alternatively, artificially stimulate TSH levels using recombinant human TSH (rhTSH) injections. 

 After administration of the radioiodine to the subject and distribution throughout the body initial exposure rates can range from 100-300 Sv.hr^-1^ at 1 m distance. A common limit for discharge is 25 Sv.hr^-1^ at 1 m which is thought to approximately equate to 600 MBq of ^131^I remaining in the average subject. Lower amounts of radioiodine used in the treatment of hyperthyroidism are usually exempt from the need for isolation and admission to a secure facility. Some jurisdictions specify a minimum isolation time in hospital while others set limits on the exposure rate from the subject for them to be discharged. The latter allows for a greater degree of flexibility as to when the subject is permitted to return to the community. 

 Temporal information about radioiodine retention in the body after ablative therapy would be helpful in better planning the subject’s discharge time. A previous study used a ceiling mounted detector to sample the whole-body retention of post-ablation radioiodine subjects at a number of discrete time points (0.5 hrs post-administration, at 2 hrs and then every 12 hrs until discharge) (2). The subjects received a warning alarm, which signalled that they should lie on the bed prior to each measurement so that the geometry of measurement was repro-ducible. They found an effective retention half time, t_½_ (Eff), of 10.5 hrs in 36 patients who underwent rhTSH stimulation prior to therapy. 

 In a further group of subjects who underwent T4 withdrawal (n=236) they found a mean retention half time of 15.7 hrs. A study using ceiling mounted GM detectors, such as we have used, made measurements at a number of discrete time points during the subjects’ admission to the isolation room in the hospital and compared this with measurements on the gamma camera (3) . A more recent report by Kääriä *et *al using a system similar to the one that we have developed used a 1 min sampling interval over the duration of admission to the hospital and found a median t_½_(Eff) of 12.6 hrs (4).

 At our institution subjects receiving 1 GBq of ^131^I are usually treated in the afternoon and discharged the following morning, those receiving 2-4 GBq are treated in the afternoon and released on the morning of the second day after treatment, and those receiving 6 GBq are usually treated on a Friday afternoon and released on Monday morning, *i.e.*, on the third day after treatment. This can vary at the discretion of the radiation safety staff provided the subject will not breach any radiation exposure guidelines with members of the public. Some subjects can be anxious about being in an isolation room for an extended period and request an early discharge when still exceeding the guidelines for maximum radiation exposure levels. Other therapies requiring isolation at our site include [^131^I]-MIBG for paragangliomas and phaeochromo-cytomas and potential clinical trials using ^131^I as the targeted radionuclide therapy.

 We have developed a continuous radiation detector monitoring system for our isolation rooms on the ward to provide exposure rates in real time after radionuclide therapy. The aim of this publication is to describe the system that has been developed and report the results for whole body retention in 250 individuals treated at our site.

## Methods

**Figure 1:**
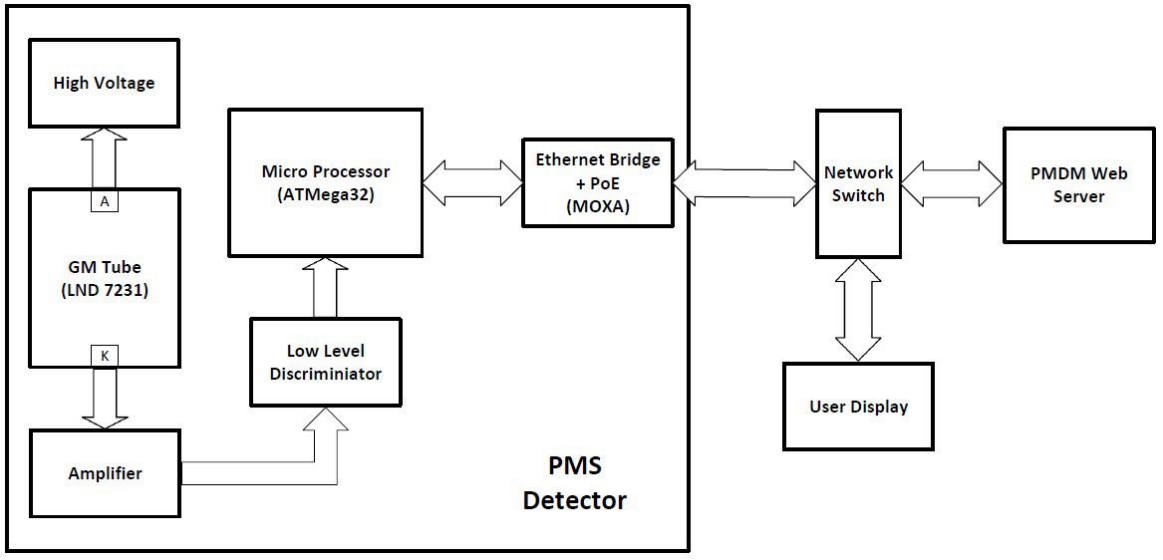
Schematic of the detector unit and connection to the web server running the software

 The radiation monitoring device that has been developed (AustralRad PMS, Globalsonics, Sydney, AUS) is based on a small Geiger-Müller (GM) tube (LND7231, Lind Inc, NY, USA) weighing 30 g and includes an internal microprocessor. It is encased in a commercially available plastic housing, which resembles a domestic smoke alarm and is mounted on the ceiling in each of our two isolation rooms that have lead-lined walls for shielding. Also in the detector housing are the electronics and ethernet communications module. The total weight of the unit is 150 g. The system communicates and receives all power (12 VDC) via an ethernet cable (Power-over-Ethernet, PoE) and thus only one cable is required for both powering and communicating with the unit (Figure 1). 

**Figure 2:**
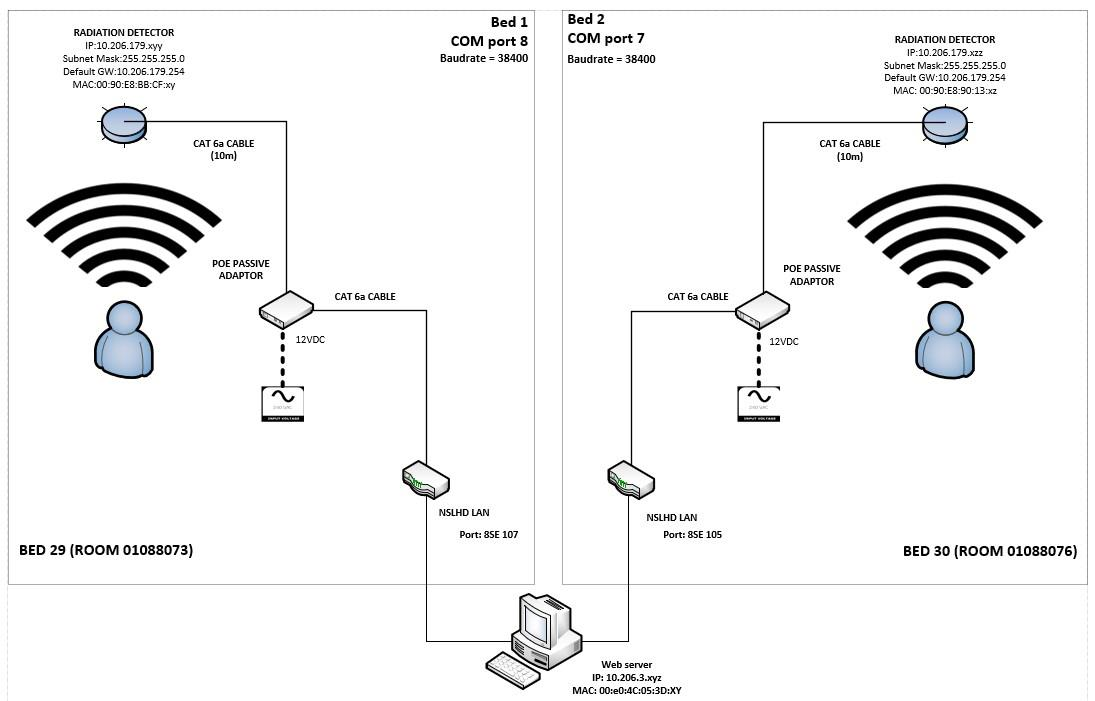
A schematic of the installation on the institutional local area network (LAN). The detectors are in the isolation rooms on the 8th floor of the main hospital building while the web-browser software that monitors the detectors is located on a single-board computer in the nuclear medicine department on the 2nd floor of the same building

 The detector is positioned directly above the hospital bed in the room, a distance of approx. 1.8 m. The ethernet cable is connected to the institutional local area network (LAN) and the detectors have static IP addresses assigned by the hospital IT department based on their individual MAC (Media Access Controller) identifiers. The cabling from each unit is connected to a pre-existing wall-mounted ethernet port on the LAN. Thus, the installation of the devices only required a dedicated cable to be installed from the ceiling-mounted detector to the standard ethernet port already connected to the network in the room. A schematic of the connections is shown in Figure 2. 

 The two rooms with the detectors are on the 8^th^ floor of our main hospital building in an Oncology ward while the software monitoring the detector outputs is run on a single-board computer (LattePanda Alpha, DFRobot, Shanghai, China) that is installed in the Department of Nuclear Medicine on the 2^nd^ floor of the same building and uses the open source Ubantu Linux operating system. The digital “count” data that are output continuously from the detectors is received by a browser-based software application developed in-house in Python. We have chosen to integrate the data over a one-minute interval and provide an average count/exposure rate per minute, although different integration times can be selected. As the software is built to run in a web-browser it does not require special software to install – the viewing computer only requires access to the PC running the browser software on the LAN. The browser software contains a database where basic information about the subject treatment is entered and then records the data after detector monitoring is activated. This then runs continuously over the entire period of the subject’s admission to the isolation room. 

 The data are written to disk on the mini-PC and stored in a comma-separated values (.csv) format which is compatible with common numerical software applications such as Microsoft Excel and are stored as two columns containing data & time in column 1 and the average measurement over the sampling interval (1 min in our case) in column 2.

 The detectors were calibrated to display accurately in Sv.hr^-1^ at 1 m using a ^137^Cs source prior to installation but at this stage no compensation has been included to change the reading to allow for the different distance from the subject lying on the bed to the detector (1.8 m) and to modify the reading for a distributed source volume rather than a point source as used for the calibration. However, the relative exposure rates provide information about the radioiodine retention allowing the analysis of the retention over time. As will be seen, as the subject moves freely about the room and leaves the room temporarily to use the *ensuite* bathroom there are discrete, stepwise changes in the measured exposure rates. To measure the overall retention half-time we have fitted a mono-exponential function to the entire data set using in-house Python scripts and extracted the time constant in hours. All data points were assigned equal weighting.

## Results

 The data that have been analysed were recorded at our single site with two isolation rooms between February 2019 and September 2020. In this time, just over 300 subjects were treated with 250 having sufficient data to be evaluable in this study. Subject demographics are contained in Table 1. 

**Table 1:** Subject demographics, pathology, treatment-related data and average retention measurements

**Parameter**	**Value**
Total Evaluable Subjects	250
**Cohort Profile**	
Female	66%
Mean age (yrs)	50.7
Min age (yrs)	17
Max age (yrs)	84
**Pathology**	
Papillary	72.8%
Follicular	13.0%
Hurthle Cell	8.6%
Mixed Papillary & Follicular	5.6%
Other	<1%
**ATA Grade**	
Low	16.2%
Intermediate	56.5%
High	27.3%
**Amount I-131 Administered**	
1 GBq	11.7%
2 GBq	8.0%
4 GBq	62.3%
6 GBq	17.9%
**Effective Half-Retention Time (hrs)**	
Mean	11.9
SD	3.2
MIN	5.0
MAX	23.1

 Recombinant human TSH stimulation was used in over 93% of subjects prior to treatment in the dataset analysed. The files produced by the monitoring software contain between 1,000 – 4,000 sampled time points depending on the duration of the subject’s admission as an inpatient. The mean t_½_ (Eff) over all subjects was found to be 11.9±3.2 hrs with a range of 5.0–23.1 hrs. We further examined the data for any age dependency in a subset of 184 subjects where we had reliable age data. Subjects were categorised according to the age cut-off of 55 years as suggested in the 8^th^ Edition of the AJCC Cancer Staging Manual (5). A univariate *t*-test was applied. As shown in Table 2, greater age was independently associated with a significantly longer t_½_ (Eff).

**Table 2:** Age-related differences in retention half time by univariate analysis

**Parameter**	**< 55 years (n=103)**	** 55 years (n=81)**	**P value**
Age (years)	40.0±0.9	68.0±7.5	<0.0001
t_½_(Eff) (hrs)	11.5±3.0	14.4±7.4	0.0007

**Figure 3:**
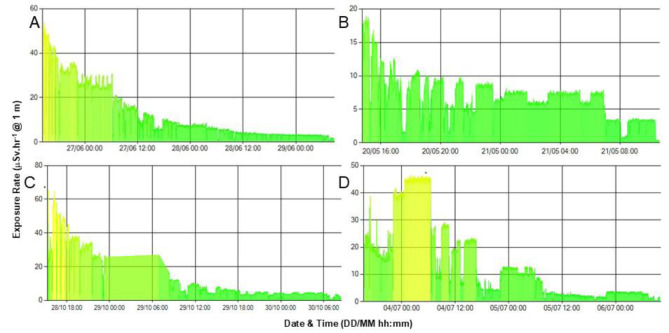
Example retention curves using the observed exposure rates in four different individuals. The colour of the data lines changes at different preset exposure levels corresponding to High (red – not shown), Intermediate (amber) and Low (green, <40  Sv.hr^-1^) exposure rates. See Results section for detailed description of the curves and their characteristics. The administered amounts of radioiodine and admission durations in these subjects were (**A**) 6 GBq for 3 days, (**B**) 1 GBq overnight, (**C**) 4 GBq for 2 days and (**D**) 4 GBq for 3 days. This is reflected in the different initial exposure rates and number of data points

**Figure 4:**
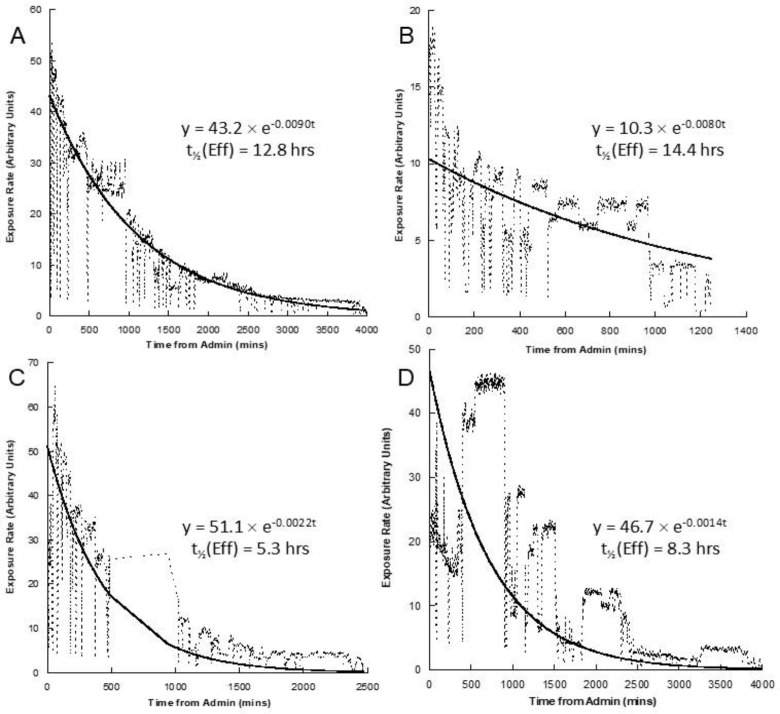
Retention curves from Figure 3 with the derived fits for each shown along with the effective retention half times. Each graph (A-D) corresponds to the data shown in the same position in Figure 3

Figure 3 shows examples of retention profiles from four treated individuals and Figure 4 shows the mono-exponential fits derived for each of the subjects in Figure 3. The effective half times for the subjects shown range from 5.3–14.4 hrs. A number of distinct patterns can be discerned in these profiles. In general, the highest sections in any period relate to time where the subjects are lying on the hospital bed, directly under the detector and hence at the closest distance to the device. This is frequently observed at night when the patients are sleeping and is frequently seen between midnight and 6:00 am. There are other prolonged periods at a slightly lower exposure rate, which correspond to when the subjects are seated in a recliner chair in the room, which is further away from the detector. This is often observed during daylight hours. Further, there are discrete stepwise decreases in the data, which likely correspond to when the subjects empty their bladders of radioactive urine or faeces in the *ensuite *bathroom. Finally, there are longer times of very low exposure which return back to the previous level likely corresponding to a prolonged period in the adjoining bathroom such as when showering.

 The profile in Figure 3(A) shows a relatively well-behaved monotonically diminishing exposure rate which is easily fitted with a mono-exponential retention curve. It should be noted that none of the retention profiles are decay-corrected as it is total radioactivity remaining in the subject which we wish to measure. Figure 3(B) is more representative of the typical profile seen. The discontinuities represent the subject moving around the room more than the subject in Figure 3(A), as displayed by the large step changes in the exposure rates and can be seen to spend prolonged periods in different locations. There is a large decrease in the measured values at approx. 07:00 hrs on 21/05 with the curve never returning to the pre-07:00 hrs level and is likely to represent the voiding of a large amount of urine containing ^131^I after waking. At approx. 08:00 hrs the measurements again drop for a period likely to correspond to the subject showering in the adjoining bathroom and hence at a greater distance from the detector. Figure3(C) displays a generally decreasing curve but with a prolonged “plateau” between approx. 23:00 hrs on 28/10 and 06:00 hrs on 29/10, presumably when the subject was asleep in the bed. Figure 3(D) is more challenging to characterise but daytime and nighttime periods generally correspond to measurements indicative of spending periods in the hospital bed during the night and moving to other locations in the room during daylight hours.

**Figure 5:**
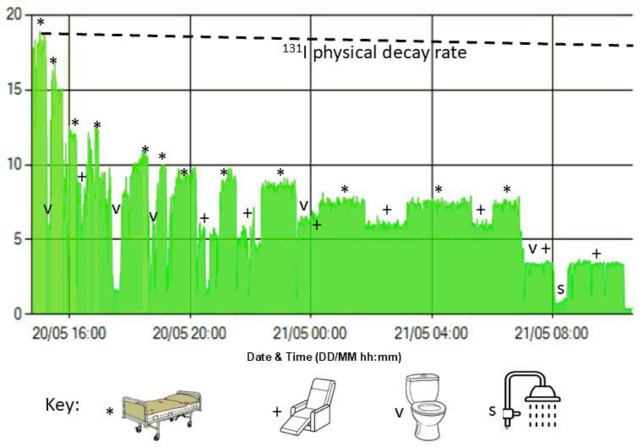
The retention profile shown in Figure 3(B) with annotations suggesting possible explanations for the observed discontinuities in the measurements as the subject goes about daily activities after the treatment administered. The symbols correspond to the activity or location assumed in the profile. The natural decay rate for 131I is shown as well. Over the period sampled there would be approx. 8% decrease in the observed exposure rate due to radioactive decay alone

Figure 5 shows the example seen in Figure 3(B) with annotations suggesting possible explanations for the discontinuities in the observed retention profile as the subject moves from the bed (*) to the recliner chair next to the bed by the window (+), uses the toilet thus producing a stepwise decrease in the observed exposure rate (v) and spends a longer time in the *ensuite *bathroom having a shower prior to their scan, as instructed, before being discharged (s).

## Discussion

 Radioiodine treatment of thyroid disorders is amongst the earliest uses of nuclear medicine, dating from the work of Saul Hertz in the 1940s (6). Hertz utilised the natural affinity of the thyroid to incorporate iodine into thyroid cells to produce thyroid hormones as an ideal way to deliver an internal radiation treatment, rather than using an external source, to an overactive thyroid and to certain thyroid cancers where the cancer cells retain their ability to extract iodine from the systemic circulation. As radioiodine is used for both imaging the subject for staging and monitoring as well as treating the subject it is considered the first radiotheranostic agent (7). As iodine-131 emits both beta particles and gamma photons, it can be used for both applications in theranostics. 

 However, a downside to this dual property is that, when ^131^I is administered in large amounts for therapy, the level of radiation emitted by the subject may be hazardous to members of the public and thus the subjects are usually admitted to the treating facility and kept in isolation for 1-5 days. Knowledge about the whole-body retention of radioiodine after ^131^I ablation for thyroid cancer is useful in helping plan for isolation with a hospital admission and may allow the subject being treated to better prepare for the peri-therapeutic period. 

 Earlier temporal measurements of radiation exposure rates after radioiodine ablation have used a discrete, small number of measurements (2, 8, 9). Similar to the methods recently reported by Kääriä et al and Chuah et al (4,10) we have developed a device that allows us to monitor the exposure rate continuously which, amongst other things, allows us to better predict when the subject can be discharged from the hospital. Continuous monitoring also provides an insight into the retention and elimination of the radioiodine by the body. 

 The main aim of this development was to be able to monitor the subjects’ retention profiles in real-time to assist in planning for the amount of time that an individual subject would need to remain isolated from the general public. Thus, it is the overall retention profile over the total duration of the admission to the isolation rooms that we are interested in rather than accurate measurements of the piecewise curves. 

 In Figure 4 the overall fit to the data can be seen to depart from the actual data in numerous cases but the overall curve does provide a single figure estimate that is useful for generally characterising the retention over the extended period of time. Stepwise discontinuities mean that the amount of radioiodine remaining in the body does not decrease in a monotonically well-behaved manner but, taken over many hours, can be used as a reasonable first approximation. 

 While we have not actually verified the causes for the variations in measurements over the duration of the inpatient admission, and have only offered possible explanations as to the reasons for the step changes, it does not have significant impact as it is the overall retention profile from the time of administration of the therapy to the time of discharge that we are interested in. For this purpose, the curves approximate the observations adequately. A further use of this will be in a clinical trial we are about to participate in which is using ^131^I radiolabelled to a LAT1 receptor target to treat primary brain tumours and for which the retention profile is largely unknown. The ability to continuously monitor the retention profile will give further insights into the response of the body to the therapeutic product.

 For this prototype we have not included some features which have been discussed and might be considered desirable. Inspection of the curves, which contain thousands of measurements, demonstrate discrete discontinuities at times corresponding to the subject moving around inside the isolation room and adjoining bathroom. This clearly provides data which are inconsistent over the duration of the admission. 

 We considered using a further device to only record data when the subject was lying in the bed, such as a pressure pad that would activate only when the subject’s weight was on the mattress. An alternative idea was to use a small temperature sensor within the monitor on the ceiling to detect when the subject was in close proximity. To date, however, we have not implemented either. A non-hardware-based alternative could be to acquire the data as we do now and use an Artificial Intelligence (AI) or Deep Learning (DL) algorithm to “clean up” the data by identifying those stepwise movements in the data to either remove them or “restore” them to the equivalent measurements derived when the subject is in bed. After initial pilot testing by removing some sections of the data when the subject was not in bed it was found that the curve fitting was barely affected as the overall pattern of retention was dominating the calculation of the half-clearance time. 

 Therefore, for this work we have not removed or modified the data in any way and are using the raw data that were produced by the detector with no further signal processing.

 Another feature that we would like to add would be to calibrate the retention curves to give accurate exposure rate measurements. While the detectors are calibrated in the factory using a ^137^Cs point source, both the varying distance from the radioiodine subject to the detector and the distribution of the radioiodine throughout the body means that the readings obtained will not necessarily be accurate. 

 Empirical corrections (*e.g.*, 1/d^2^ for distance correction) for both could be applied but are still unlikely to give an accurate measurement, even in the situation where the subjects are lying on the bed, given the differences in individuals’ body size, shape and biodistribution. One approach could be to have a staff member take a reading with a hand-held radiation monitor at a distance of 1 m from the treated subject an hour after the radioiodine was administered, when it is becoming distributed more widespread throughout the body, and use this as a parameter that is input to the monitoring software to give the system a “starting value” for the exposure rate at 1 m at that point in time, with all other subsequent measurements referenced to the initial one.

 The installation of this system is very simple and similar to installing a ceiling-mounted domestic smoke detector. The only addition is that a dedicated Ethernet cable is installed in the ceiling and wall space so that it can connected to the institutional LAN. All data from the detector are transmitted over the LAN to the monitoring software on the web-browser, which can be viewed from any device connected to the LAN as it is browser-based and therefore does not require dedicated software to be installed to view the graphical data that are displayed “on the fly”. One potential application is to display the retention curves, such as seen in Figure 3, in real-time on the monitors already installed in the anteroom outside the isolation rooms to provide colour-coded visual feedback to the nursing and domestic staff to provide reassurance as to when it is safe for them to enter the room with the subject.

 The mean half-clearance time that we have measured (11.9±3.2 hrs) in subjects who have received rhTSH stimulation is similar to that reported by Remy *et al* (10.5±1.5 hrs) who based their estimate on a smaller number of sample points (5-7) as they made a measurement only every twelve hours after initial measures at 0.5 and 2 hrs. Also similar to their findings, we demonstrate an age dependency using a cut-off of 55 years. 

 The median retention half-life reported by Kääriä et al of 12.6 hrs also closely matches our observations (4). A retention half time of around 12 hrs is well suited to our current clinical practice of hospitalising subjects treated with 2, 4 or 6 GBq of radioiodine for 40-66 hours. After 40 hrs the mean retention rate constant (=0.058 hrs^-1^) equates to, on average, 10% (*e*^-40^) of the initial administered amount of radioiodine remaining in the body, thereby fulfilling our guidance criteria of discharging the subject with less than 600 MBq of radioiodine remaining *in vivo*.

## Conclusion

A device has been developed that provides continuous monitoring of radioactive emissions from subjects having diagnostic or therapeutic procedures using radiopharmaceuticals. 

 Installation is simple and utilises the institutional LAN infrastructure to communicate with the monitoring software at a remote location. We have used it to examine the retention half time in 250 subjects treated for thyroid cancer with ^131^I after rhTSH stimulation and find an average value of approx12 hrs.
